# Central retinal vein and artery occlusion associated with sildenafil: a case report and review of the literature

**DOI:** 10.1186/s13256-023-04104-8

**Published:** 2023-09-20

**Authors:** Ali Torkashvand, Pasha Anvari, Siavash Ketabi, Esmaeil Asadi Khameneh

**Affiliations:** 1 Farabi Eye Hospital, Tehran University of Medical Science, Tehran, Iran; 2 Eye Research Center, Five Sense Health Institute, Tehran, Iran; 3 Iran University of Medical Science, Tehran, Iran; 4 Tehran University of Medical Science, Tehran, Iran

**Keywords:** Central retinal artery occlusion, Central retinal vein occlusion, Sildenafil

## Abstract

**Background:**

Sildenafil is a selective phosphodiesterase type 5 inhibitor used for the treatment of erectile dysfunction and pulmonary hypertension. It is available over the counter in many countries. While there have been a few reports of retinal vascular occlusion following sildenafil consumption, most cases have other comorbidities as risk factors for the disease, and the exact causal role of this drug in these conditions remains unclear.

**Case presentation:**

We present the case of a healthy 32-year-old Iranian man who developed combined central retinal vein occlusion and retinal artery occlusion following sildenafil exposure. The patient underwent a hypercoagulative state workup for possible underlying risk factors. Additionally, we conducted a literature search on PubMed using the keywords: retinal vein occlusion AND Sildenafil OR Viagra, retinal artery occlusion AND Sildenafil OR Viagra, retinal vascular occlusion AND Sildenafil OR Viagra. To obtain more objective results in the reviews, we employed an adverse drug reaction possibility algorithm. The patient was found to be otherwise healthy, and ancillary tests were unremarkable. A literature review identified seven reports of retinal vascular occlusion following sildenafil use. In most of these cases, the role of sildenafil was not clearly established. To the best of our knowledge, our case achieved the highest score based on the algorithm compared with previous reports.

**Conclusion:**

Sildenafil may be associated with severe retinal vascular accidents in otherwise healthy young individuals.

## Background

Sildenafil is a selective phosphodiesterase type 5 (PDE5) inhibitor approved for erectile dysfunction (ED) and pulmonary artery hypertension (PAH) [1]. It induces vasodilation by relaxing smooth muscles of arterioles through increasing intracellular cyclic guanosine monophosphate (cGMP) [2].

Retinal toxicity and side effects of sildenafil, including transient blue/green vision, blurred vision, and photosensitivity [3], have been attributed to a minor inhibitory effect of sildenafil on phosphodiesterase type 6 (PDE6) [4]. The drug’s effect extends beyond visual disturbance to affect the hemodynamics of the retina and choroid [5, 6]. Several studies have reported retinal vein or artery occlusion and non-arteritic anterior ischemic optic neuropathy (NAAION) following sildenafil treatment, where most reported cases had other risk factors for vascular accidents. In this study, we report a young man with combined central retinal vein and artery occlusion without underlying thrombophilia or systemic cardiovascular risk factors.

## Case presentation

**Fig. 1:**
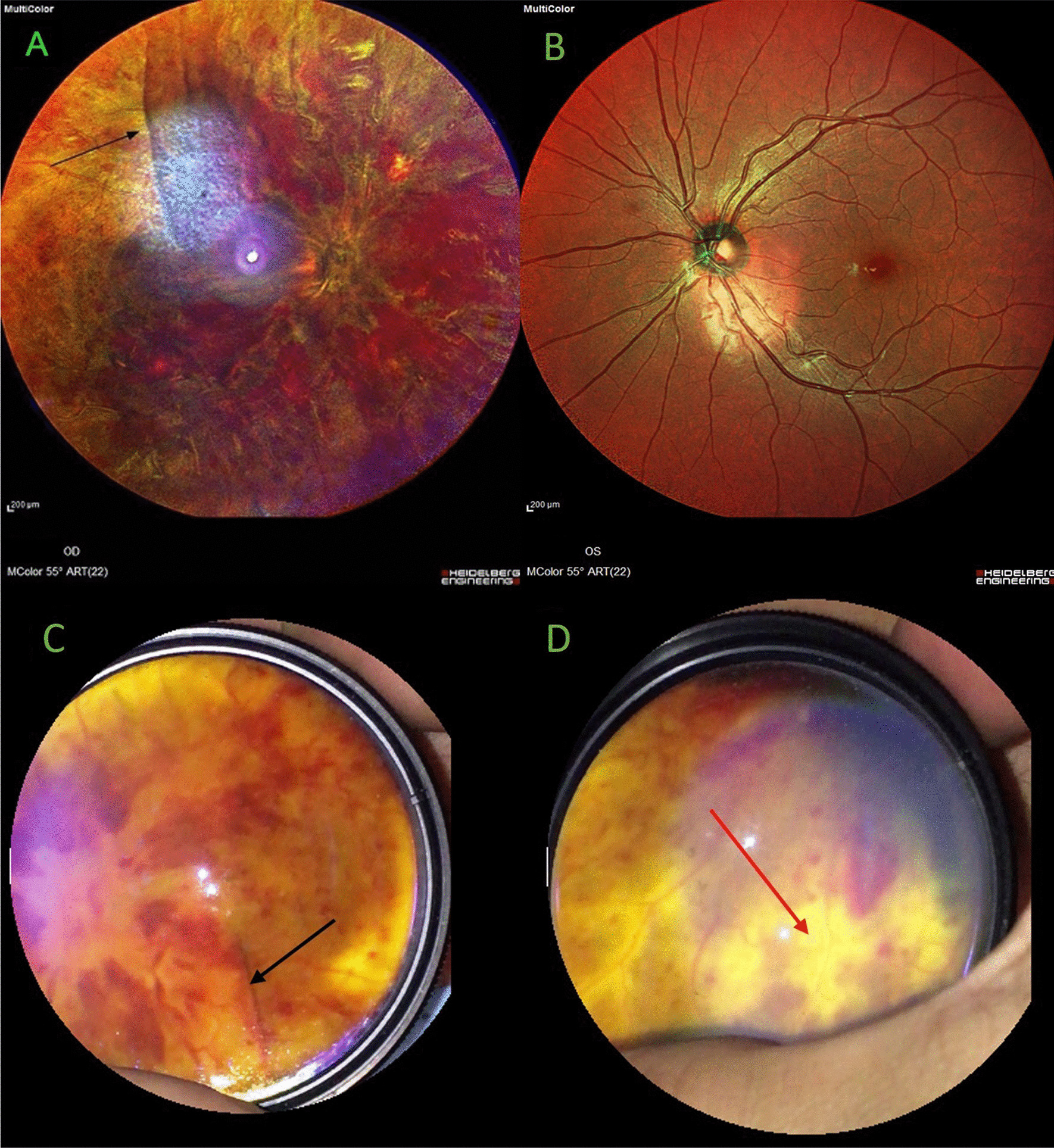
**A** Multicolor fundus photograph of right eye displaying pronounced disc swelling and extensive retinal hemorrhage. The black arrow indicates the macular fold from the severe retina and optic disc swelling. **B** Normal left eye with a normal cup–disc ratio. **C**, **D** Images captured by indirect mobile ophthalmoscopy, inverted and flipped. The macular fold is well appreciated (black arrow), and subretinal exudation is clearly visible in the peripheral view (red arrow)

**Fig. 2:**
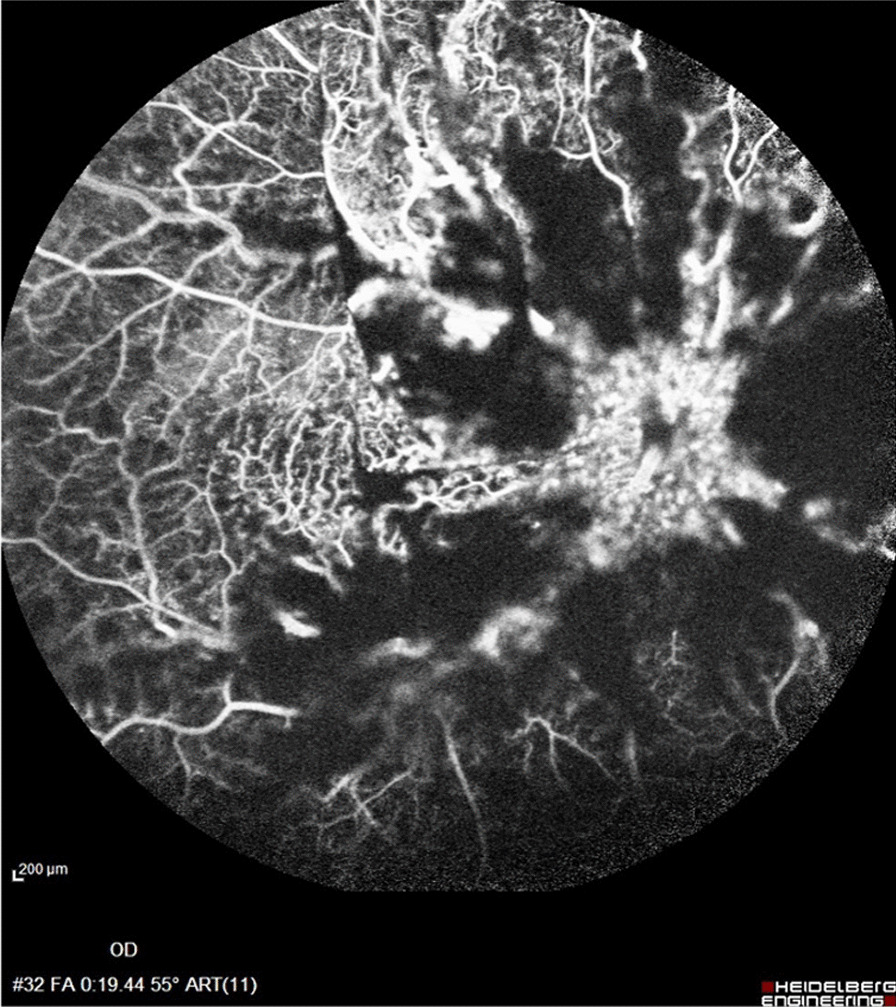
Fluorescein angiography reveals blockage of the fluorescein due to severe retinal hemorrhage and vascular staining

A 32-year-old Iranian healthy man presented to us with sudden vision loss in the right eye 3 hours following 100 mg sildenafil consumption. The history for thrombophilia, diabetes mellitus, and hypertension was negative. No familial history of hypercoagulative diseases was noted. The patient reported no vigorous exercise and severe dehydration. Visual acuity was 20/200 OD and 20/20 OS. Intraocular pressure (IOP) was 12 mmHg OD and 14 mmHg OS. Ophthalmic examination revealed a three-plus positive relative afferent pupillary defect (RAPD) in the right eye. In fundus examination, a diffuse retinal hemorrhage, cotton-wool spots, subretinal exudation, dilated and tortured veins, and prominent optic disc swelling were evident. The extreme severity of exudation and retinal edema resulted in macular folding (Fig. 1A, black arrow). Examination of the contralateral eye was unremarkable, and the cup–disc ratio was normal (Fig. 1B). Fluorescein angiography depicted blockage of the fluorescence due to extensive retinal hemorrhage and peripheral retinal hypoperfusion, and vascular staining, probably due to ischemia, was appreciated (Fig. 2). Due to clinical examination, severe peripheral nonperfusion, and occluded retinal arterioles in periphery, the diagnosis of combined central retinal vein occlusion and central retinal artery occlusion was made for the patient. Since recently, a few cases of retinal vascular accidents following severe acute respiratory syndrome coronavirus 2 (SARS-COV-2) have been reported; we asked him about his recent history of coronavirus disease 2019 (COVID-19) or recent vaccination, which were negative.

To rule out undetected underlying systemic disease, we conducted ancillary laboratory tests oriented to hypercoagulable states including the level of serum protein–S &–C, homocysteine of the serum and urine, antithrombin–III and factor V–Leiden, where none of the tests were significant. The plasma level of sildenafil was not measured because the test was not available. The patient was treated with intravitreal bevacizumab (Avastin) injection for severe macular edema and hemorrhage. After three monthly intravitreal bevacizumab injections, the macular edema resolved but the vision did not improve due to severe macular ischemia and optic disc atrophy. To assess the ADR probability, we implemented the Naranjo algorithm (Table 1) and the scores for each item related to our case is bolded.

**Table 1:** Naranjo algorithm for assessment of probability of adverse drug reaction (ADR)

Question	Yes	No	NA
1. Are there previous conclusive reports on this reaction?	+ 1	**0**	0
2. Did the adverse event appear after the suspected drug was administered?	** + 2**	−1	0
3. Did the adverse event improve when the drug was discontinued or a specific antagonist was administered?	+1	**0**	0
4. Did the adverse event reappear when the drug was readministered?	+2	−1	**0**
5. Are there alternative causes that could on their own have caused the reaction?	−1	** +2**	0
6. Did the reaction reappear when a placebo was given?	−1	+1	**0**
7. Was the drug detected in blood or other fluids in concentrations known to be to	+1	0	**0**
8. Was the reaction more severe when the dose was increased or less severe when the dose was decreased?	+1	0	**0**
9. Did the patient have a similar reaction to the same or similar drugs in any previous exposure?	+1	0	**0**
10. Was the adverse event confirmed by any objective evidence?	+1	0	**0**
Total score	Interpretation of scores		
Total score ≥ 9	Definite		
Total score 5–8	Probable		
Total score 1–4	Possible		
Total score ≤ 0	Doubtful		

The adverse drug reaction probability scale consists of ten questions that are answered as either “Yes,” “No,” or “Do not know.” Different point values (−1, 0, +1 or +2) are assigned to each answer. The score of our case is bolded

## Discussion

Sildenafil is a potent PDE5 inhibitor with a weak PDE6 inhibitory effect that is generally prescribed for ED and PAH; in many countries, it is available at pharmacies without physicians’ affirmation. The effects of sildenafil on the eye are usually divided into two groups: the dose-dependent effect of PDE6 inhibition on photoreceptors by controlling the level of cGMP that causes reversible blurred vision, blue–green tinge vision, light sensitivity, and decreased color vision, and the effects that are results of PDE5 inhibition; most of these are related to change in vascular structure and the coagulation system that can induce serious and irreversible retinal and optic nerve catastrophes. The effect of sildenafil on retinal and optic disc blood flow is controversial. Grunwald [7] showed that perfusion of the optic nerve and choroid are not affected by sildenafil, but there are numerous studies that indicate choroidal perfusion increases, while retinal vascular flow remains unchanged [8]. The discrepancies between studies are presumably due to variability in measurement methods of the vascular flow and different protocols of the studies and participants. Particularly in the deeper vascular layer of the optic nerve, some measurements may have not enough validity [9, 10]. Several serious retinal vascular accidents have been reported following sildenafil use [11–17], but the exact role of this medication is not clear.

We reported a 32-year-old healthy man with central retinal vein occlusion (CRVO) and retinal artery occlusion (RAO), 3 hours following 100 mg sildenafil use. Since the patient was otherwise healthy without known risk factors for vascular disease, negative ancillary test results directed to hypercoagulative states, and in addition the close temporal relation between sildenafil intake and incidence of the disease, we speculated that the drug could play a causal role in this clinical scenario.

We employed the Naranjo algorithm [18] (Table 1), for the assessment of the probability of the adverse drug reactions (ADRs). However, these algorithms have some inherent limitations such as semiquantitative measurements and arbitrary weight of scores. In addition, some of the items are not applicable to certain conditions [19], for instance, we are not allowed to do rechallenge tests in severe conditions, or some of the ADRs are not reversible after drug withdrawal and some ADRs are idiosyncratic and do not have dose–dependent relationship. Here we review the previously reported cases of retinal vascular accidents following sildenafil usage with critical scrutiny (Table 2).

**Table 2:** Previously reported cases of retinal vascular accidents following sildenafil usage with critical scrutiny

Study	Accident	Age	Time to event	Comorbidity and risk factors	Sildenafil dose	Score
Current study	CRVO, NAAION, BRAO	32	3h	NO	100	4
Tripathi [16]	BRAO	69	4	NA	100	2
Bertolucci [21]	Hemi-RAO	51	4	HTN	100 + 100 (1 day before)	1
Akash [12]	NAAION, Cilioretinal artery occlusion	54	few	No	200	2
Gedik [13]	CRVO NAAION Cilioretinal artery occlusion	36	< 24h	Hemodialysis Small cup/disc	100	1
Pinto [20]	CRVO	74	72h	COPD, PAH	NA	3
Hafidi [15]	CRVO, Cilioretinal artery occlusion	40	NA	NA	100 + 100 (on two consecutive days)	2
Sinha [22]	NAAION, BRVO	62	NA	Smoking, small cup/disc ratio	200	1

*CRVO* central retinal vein occlusion,* NAAION* non-arteritic anterior ischemic optic neuropathy,* BRAO* branch artery vein occlusion,* Hemi-RAO* hemi-retinal artery occlusion,* HTN* hypertension,* COPD* chronic obstructive pulmonary disease,* PAH* pulmonary artery hypertension,* BRVO* branch retinal vein occlusion

Retinal vein occlusion (RVO): In 2007, Gedik *et al.* reported a 36-year-old man with CRVO, NAAION, and cilioretinal occlusion following sildenafil use [13]. The patient was undergoing hemodialysis for chronic kidney disease (CKD), a well-known risk factor for vascular accidents. Additionally, the contralateral eye had a small cup/disc ratio, which is a significant risk factor for NAAION. In the correspondence by Oguz [11], it is stated that the disease is unlikely to occur secondary to sildenafil use since the patient had other significant risk factors for the disease. However, it is notable that the patient had presented with hemifield loss in the contralateral eye 3 months later following sildenafil consumption despite the warning on the medication. Although the patient was under treatment for CKD and had other remarkable risk factors for the disease, two episodes of the disease after sildenafil consumption (rechallenge) and the presence of a close temporal relation raise this suspicion that sildenafil could be responsible for the event. Furthermore, it is crucial to mention that the disease may be multifactorial, and a cumulative pattern of risk factors could lead to the disease and the presence of risk factors does not rule out other possible causes.

This was the most similar case to ours in the literature, but our case was more severe with extensive intraretinal hemorrhage, prominent optic disc swelling, exudative retinal detachment, and retinal ischemia that extended to the equator.

Pinto *et al.* reported a 74-year-old man with pulmonary hypertension who presented with CRVO, 72 h after taking sildenafil [20]. Hafidi *et al.* reported a case of CRVO and cilioretinal occlusion in a 40-year-old, otherwise healthy man following 2-day consumption of sildenafil [15]. While retinal vein occlusion in young adults is not common, targeted evaluation for hypercoagulative states is mandatory. However, neither Pinto *et al.* nor Hafidi *et al.* performed ancillary tests in this regard, and the “cause and effect” relation remains unclear.

Retinal artery occlusion (RAO): In 2000, Tripathi *et al.* reported the first case of branch retinal artery occlusion (BRAO) in a 69-year-old man after taking a 100 mg Viagra pill [16]. The authors noted that the patient was otherwise healthy and physical examination did not reveal any cardiac and carotid artery diseases. The causal role of sildenafil in that event remains elusive, as the authors did not perform any supplementary tests to assess undiagnosed carotid artery stenosis or cardiac disease. However, the event occurred only a few hours after taking Viagra, which suggests that the drug may have at least partially contributed to the disease.

Bertolucci *et al.* reported the case of a 51-year-old man who experienced hemiretinal artery occlusion after using sildenafil during sexual activity [21]. The authors suggested that was likely coincidental as a hyperreflective embolus was visible in the fundus and the patient had uncontrolled hypertension and carotid stenosis. They speculated that sexual activity with consequent hypertension and increased cardiac output may ulcerate the atherosclerotic plaque and this may be unrelated to sildenafil use.

Akash *et al.* documented a 54-year-old man who developed NAAION and cilioretinal occlusion a few hours following the consumption of 200 mg of sildenafil [12]. The patient had no known underlying disease and ultrasonography for cardiovascular diseases and laboratory tests for hyperviscosity disorders were insignificant. The patient underwent a temporal artery biopsy and the result was negative for giant cell arteritis. The authors suspected that sildenafil was responsible for the disease owing to the close temporal relation. They believed that this happened secondary to increased choroidal pressure simultaneous with decreased systemic blood pressure and subsequently decreased ocular perfusion.

Sinha *et al.* reported a 62-year-old man with NAAION and BRAO following an overdose of sildenafil [22]. The patient was a heavy smoker with a small cup/disc ratio, which is considered a risk factor for NAAION, therefore this event may be unrelated to sildenafil and the correlation between sildenafil and the vascular accident is not clear in this case.

In 2016, the Food and Drug Administration (FDA) reported the retinal vascular occlusions associated with phosphodiesterase type 5 inhibitor use. By the end of 2014, 82 cases of RVOs and 24 RAOs had been reported, with 32 and 12 of them having other risk factors and comorbidities, respectively. The FDA postulated that these diseases are multifactorial and recommended that ophthalmologists ask patients with retinal vascular occlusion about their PDE5 inhibitor consumption [23].

The current study reported a case that achieved a Naranjo score of at least 4, which is the highest compared with previous reports and drew attention to possible serious adverse effects of sildenafil. However, the exact mechanism of this event is not clear. One hypothesis is that, in the setting of decreased systemic blood pressure, PDE5 inhibitors induces retinal vein engorgement, and since they share a common adventitia with central retinal artery at the level of lamina cribrosa, they compress central retinal artery that eventually leads to retinal ischemia, endothelial injury, vasospasm, and even central retinal artery occlusion [24]. Second hypothesis is that sildenafil might induce disc edema secondary to NAAION as a possible side effect of PDE5 inhibitors. Then, severe edema at the optic disc induces venous stasis and consequent retinal vein occlusion. Another hypothesis is that sildenafil caused relaxation of arterioles smooth muscles and this caused subsequent dislodgement of preexisting thrombus, resulting in central retinal artery and central retinal vein occlusion. Although the carotid Doppler test was not performed in our patient, this test could help to diagnose the underlying cause after such episodes. Nevertheless, the effect of sildenafil on the retinal vascular structure is not limited to the mentioned hypotheses. Capece *et al.* showed a decreased vessel density of retina and optic nerve in the group of participants consuming tadalafil for more than 6 months compared with the matched control group, and they theorize that this medication could damage retinal capillaries [25]. The vascular effect of 5-PDE inhibitors is not limited to the retina and optic disc; there are several reports of cerebral vascular accident, myocardial infarction, and deep vein thrombosis following consumption of these medications [26]. We postulate that just a mechanical concept such as compartment syndrome at optic disc dose not explain all of the cases; rather probably a more complex mechanism that induces vascular injury plays a significant role. Noteworthily, it is common among particularly young patients to use other recreational drugs such as cocaine with sildenafil, which could lead to malignant hypertension and end-organ damage such as hypertensive retinopathy, which may present by optic disc edema and retinal hemorrhages with exudates. Therefore, it is crucial to consider this association when facing young people in this scenario. However, our case did not have any history of cocaine consumption and his blood pressure was within normal limit; further, he had unilateral disease. For this reason, cocaine toxicity was less probable.

Certainly, this study has inherent limitations that should be addressed in future investigations, specifically small sample size and a dearth of established treatment criteria for evaluating adverse drug reactions. Various algorithms for this purpose do not have a uniform scoring system, and weighting the parameters is not based on clinical possibility. Furthermore, some of these items are not applicable in all conditions. Some authors believe that these drawbacks make these algorithms less valid [19]. On the other hand, we did not measure the serum level of sildenafil as the measurement was not accessible at that point, the patient did not had other signs and symptoms of toxicity, and he had just received a standard 100 mg dose of sildenafil.

## Conclusion

We express concern regarding the possible association between sildenafil use and retinal vascular accidents, particularly among young, otherwise healthy individuals.

## Data Availability

Not applicable.

## Abbreviations

ADR: Adverse drug reaction
BRAO: Branch retinal artery occlusion
cGMP: Cyclic guanosine monophosphate
CKD: Chronic kidney disease
COVID-19: Coronavirus disease
CRVO: Central retinal vein occlusion
ED: Erectile dysfunction
FDA: Food and Drug Administration
IOP: Intraocular pressure
NAAION: Non-arteritic anterior ischemic optic neuropathy
PAH: Pulmonary artery hypertension
PDE5: Phosphodiesterase type 5
PDE6: Phosphodiesterase type 6
RAO: Retinal artery occlusion
RAPD: Relative afferent pupillary defect
RVO: Retinal vein occlusion
SARS-COV-2: Severe acute respiratory syndrome coronavirus 2
